# Supplementary material for: Aberrant Expression of COX-2 and FOXG1 in Infrapatellar Fat Pad-Derived ASCs from Pre-Diabetic Donors
Source: Cells. 2022 Aug 1;11(15):2367. doi: 10.3390/cells11152367 (PMC9367583; doi:10.3390/cells11152367)
Supplement: Supplementary file 1 [file cells-11-02367-s001.zip › cells-1635967-supplementary.pdf]

Aberrant expression of COX-2 and FOXG1 in Infrapatellar Fat Pad-Derived ASCs from Pre-Diabetic Donors

Supplementary Materials:

Results:

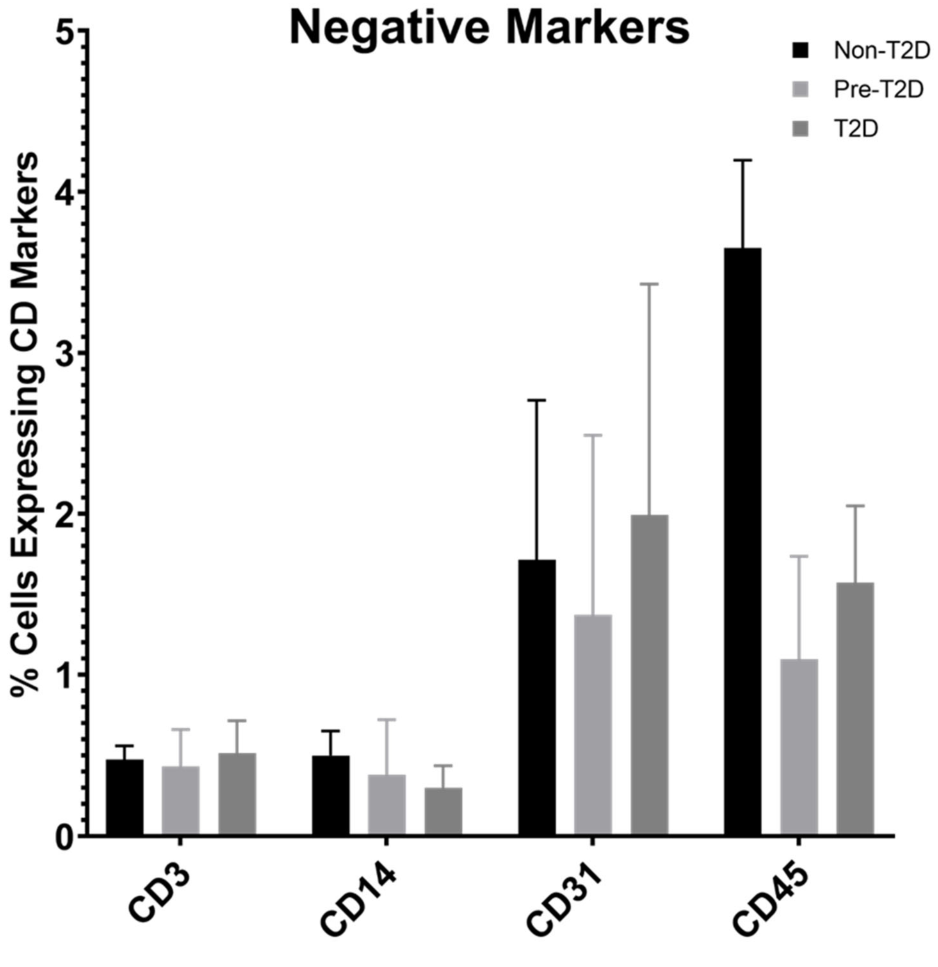

Figure S1. Decreased expression of CD90, CD73, and CD105 by Pre-T2D IPFP-ASCs.

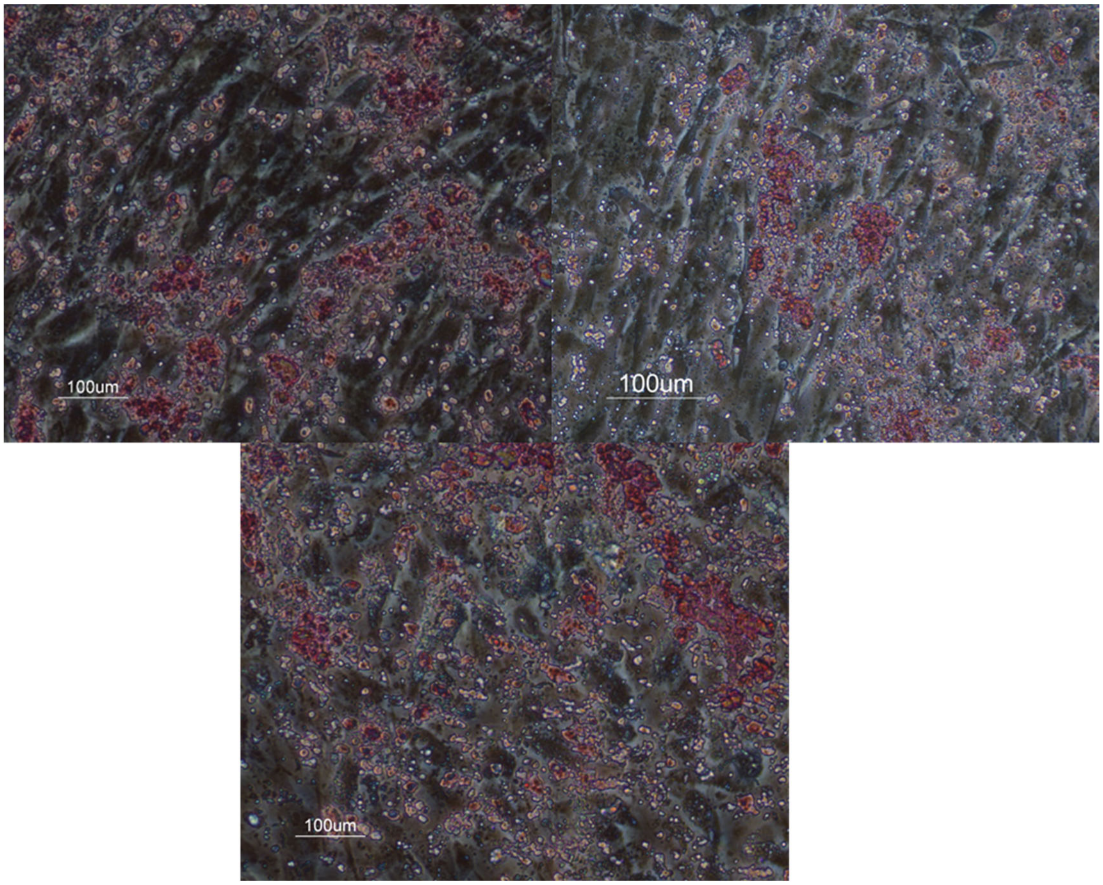

Figure S2. There was no notable differences osteogenic differentiation between IPFP-ASCS-Isolated from Non-T2D, Pre-T2D, and T2D sample groups. Alizarin Red representative image of IPFP-ASCs cultured in pro-osteogenic media for 28 days (Scale Bar: 100μm).

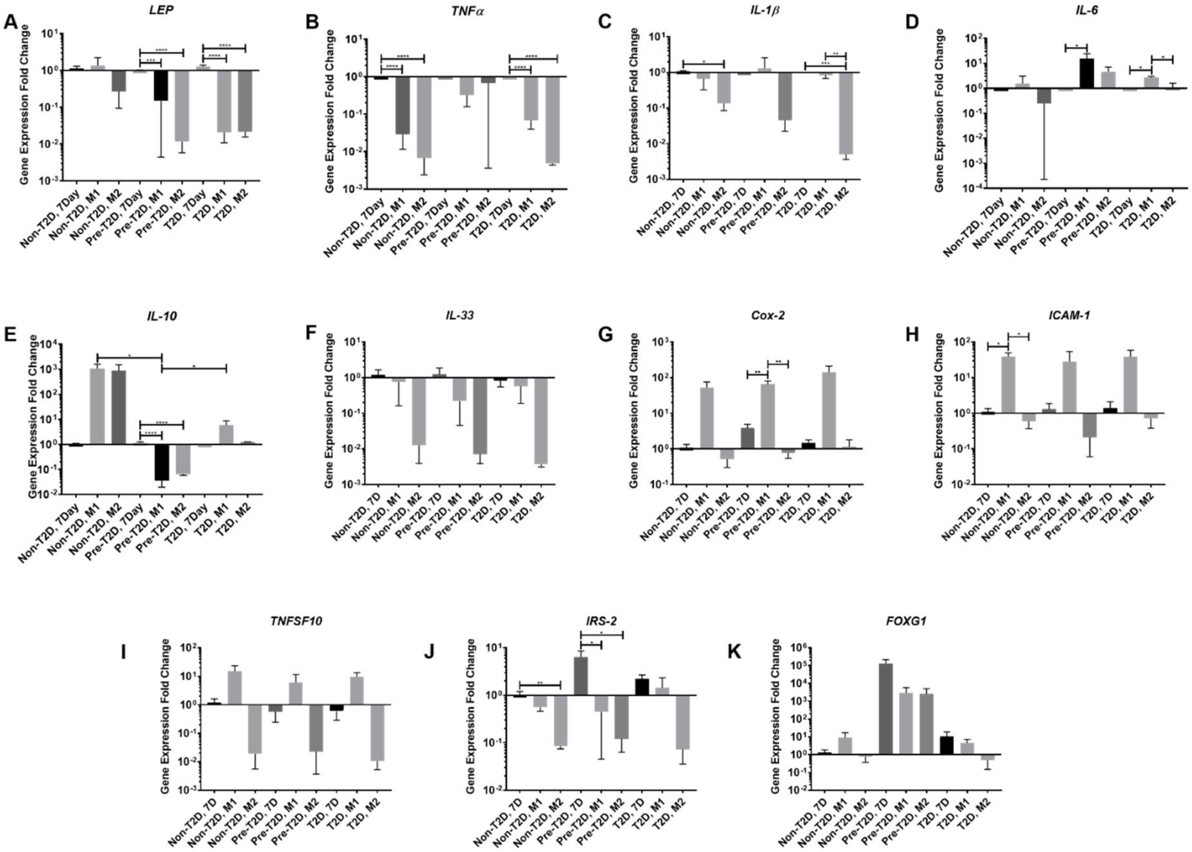

Figure S3. Decreased *TNFα*, IL-1β, and *IL-6* expression by M2 stimulated macrophages after co-culture with Pre-T2D IPFP-ASCs. (A-K) IPFP-ASC mRNA expression determined by RT-qPCR (N=3, * P<0.05, ** P<0.01, *** P<0.001). Control Group: 7Day ASCs . Non-T2D: Non-Type II diabetes mellitus, Pre-T2D: Pre-Type II diabetes mellitus, T2D: Type II diabetes mellitus.
